# Progression of Retinal Pigment Epithelial Atrophy in Antiangiogenic Therapy of Neovascular Age-Related Macular Degeneration

**DOI:** 10.1016/j.ajo.2015.02.020

**Published:** 2015-06

**Authors:** Christopher Schütze, Manuela Wedl, Bernhard Baumann, Michael Pircher, Christoph K. Hitzenberger, Ursula Schmidt-Erfurth

**Affiliations:** a Department of Ophthalmology, Medical University of Vienna, Austria, Vienna, Austria; b Center for Medical Physics and Biomedical Engineering, Medical University of Vienna, Austria, Vienna, Austria

## Abstract

**Purpose:**

To monitor retinal pigment epithelial (RPE) atrophy progression during antiangiogenic therapy of neovascular age-related macular degeneration (AMD) over 2 years using polarization-sensitive optical coherence tomography (OCT).

**Design:**

Prospective interventional case series.

**Methods:**

setting: Clinical practice. study population: Thirty patients (31 eyes) with treatment-naïve neovascular AMD. observation procedures: Standard intravitreal therapy (0.5 mg ranibizumab) was administered monthly during the first year and pro re nata (PRN; as-needed) during the second year. Spectral-domain (SD) OCT and polarization-sensitive OCT (selectively imaging the RPE) examinations were performed at baseline and at 1, 3, 6, 12, and 24 months using a standardized protocol. RPE-related changes were evaluated using a semi-automated polarization-sensitive OCT segmentation algorithm and correlated with SD OCT and fundus autofluorescence (FAF) findings. main outcome measures: RPE response, geographic atrophy (GA) progression.

**Results:**

Atrophic RPE changes included RPE thinning, RPE porosity, focal RPE atrophy, and development of GA. Early RPE loss (ie, RPE porosity, focal atrophy) increased progressively during initial monthly treatment and remained stable during subsequent PRN-based therapy. GA developed in 61% of eyes at month 24. Mean GA area increased from 0.77 mm^2^ at 12 months to 1.10 mm^2^ (standard deviation = 1.09 mm^2^) at 24 months. Reactive accumulation of RPE-related material at the lesion borders increased until month 3 and subsequently decreased.

**Conclusions:**

Progressive RPE atrophy and GA developed in the majority of eyes. RPE migration signifies certain RPE plasticity. Polarization-sensitive OCT specifically images RPE-related changes in neovascular AMD, contrary to conventional imaging methods. Polarization-sensitive OCT allows for precisely monitoring the sequence of RPE-related morphologic changes.

Age-related macular degeneration (AMD) is a progressive disease leading to substantial visual loss.^1–3^ Independent of the 2 classic pathways of disease progression with an atrophic or a neovascular development, a leading pathophysiologic role of the retinal pigment epithelium (RPE) has been recognized.^4,5^ Defects in the RPE layer continuity with abnormal choroidal vessel growth cause leakage and fluid accumulation resulting in rapid deterioration of vision^5^ owing to successive damage to the overlying retina, while clinically masking RPE morphology.

Vascular endothelial growth factor (VEGF)-A is a key factor in the pathogenesis of choroidal neovascularization (CNV).^6–8^ Milestone clinical trials have demonstrated significant efficacy in terms of improving visual acuity (VA) with monthly injections of ranibizumab. The antibody fragment inhibits binding of multiple active forms of VEGF-A to their receptors, resolves leakage, and restores retinal morphology and often function, and became the first-line treatment for neovascular AMD.^9–14^ Recently, an increased progression rate of geographic atrophy (GA) has been recognized during anti-VEGF therapy.^15,16^

Together with anti-VEGF therapy, high-resolution imaging technologies such as spectral-domain optical coherence tomography (SD OCT) that obtain high-resolution retinal images have become increasingly important modalities in the diagnosis and therapeutic management of neovascular AMD.^17^ However, current SD OCT technology visualizes retinal structures exclusively by intensity-based imaging and has substantial limitations in identifying the RPE owing to difficulties in segmenting structures of similar reflectivity. A distinct evaluation of RPE morphology would be of major relevance to gain insight into the primary pathophysiology of AMD, the biologic response to anti-VEGF therapy, and long-term prognosis.

Recently, polarization-sensitive OCT has been introduced,^18–20^ providing morphologic information beyond nonspecific back-scattered intensity patterns, selectively identifying the RPE by measuring several intrinsic tissue qualities simultaneously with spectral-domain high-resolution imaging (reflectivity, retardation, optic axis orientation, degree of polarization uniformity [DOPU]).^18–20^ Polarization-sensitive OCT provides distinct identification of the RPE condition in AMD with drusen,^21,22^ advanced dry AMD,^22,23^ and neovascular AMD.^24^ The purpose of the current study was to identify characteristic RPE changes in patients with neovascular AMD undergoing continuous anti-VEGF therapy from early to advanced changes using polarization-sensitive OCT together with conventional SD OCT.

## Methods

### Inclusion and Exclusion Criteria

Thirty treatment-naïve patients (31 eyes) with neovascular AMD were included in this prospective interventional case series. The mean age of patients was 82 (standard deviation [SD]: 8) years; 18 patients were female and 12 were male. The character and possible consequences of the study were explained in detail prior to inclusion. Each patient gave signed informed consent. Ethics Committee approval (Medical University of Vienna) was obtained. The study adhered to the tenets of the Declaration of Helsinki and to all federal laws of Austria. This study is registered at https://eudract.ema.europa.eu/, number 2006-005684-26.

Active subfoveal CNV was identified using protocol fluorescein angiography (FA) and conventional SD OCT (Spectralis OCT; Heidelberg Engineering, Heidelberg, Germany) that showed retinal thickening >250 μm. Exclusion criteria were other retinal diseases including primary GA, retinal dystrophies, and severe media opacities. All patients underwent a standardized ophthalmologic examination including best-corrected visual acuity (BCVA) using Early Treatment Diabetic Retinopathy Study (ETDRS) letters, slit-lamp biomicroscopy, fundus photography, and FA.

### Retinal Pigment Epithelium Imaging

Patients were imaged by conventional SD OCT, by fundus autofluorescence (FAF), and by a polarization-sensitive OCT prototype specifically imaging the RPE, developed by the Center for Medical Physics and Biomedical Engineering, Medical University of Vienna, used previously.^21–24^ Retinal and RPE morphology was assessed prior to anti-VEGF treatment (baseline) and at 1, 3, 6, 12, and 24 months following baseline.

Polarization-sensitive OCT measures reflectivity, retardation, optic axis orientation, and DOPU^18^ simultaneously and images retinal morphology, the RPE, and related depolarizing structures containing melanin. Details of the instrument have been published.^22^ RPE identification is based on its depolarizing quality, scrambling the polarization state of back-scattered light, while light back-scattered from other retinal layers remains in a well-defined polarization state. High-resolution 3D polarization-sensitive OCT datasets are recorded in 3.3 seconds with a raster scanning speed of 20 000 A-scans/s and 4.5 μm axial resolution. A 6.2 × 6.7 mm^2^ scanning area and a 128 B-scans × 512 A-scans scanning density was used, enabling best image quality.

**Figure 1:**
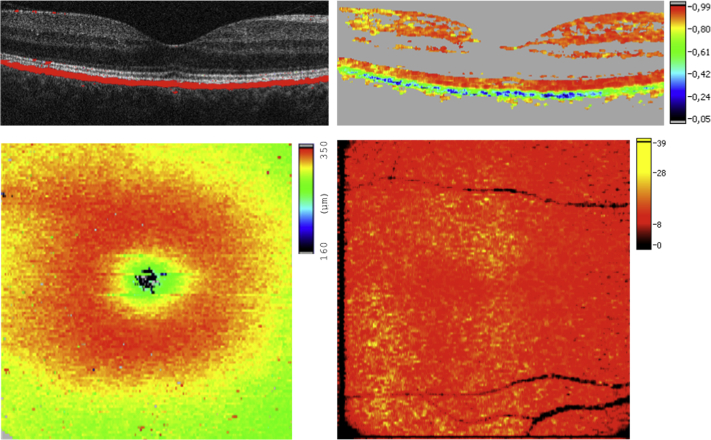
Polarization-sensitive optical coherence tomography imaging allows for selective differentiation of the retinal pigment epithelium based on intrinsic tissue-specific polarization contrast. An overlay of an intensity image and the segmented retinal pigment epithelium (RPE; red) in a healthy subject is shown (Top left). The degree of polarization uniformity (DOPU) image selectively delineates the RPE morphology (Top right). Central retinal thickness (CRT) maps may be generated (Bottom left), as well as a selective RPE thickness map (Bottom right).

RPE segmentation was performed by an algorithm based on Stokes vector analysis.^18^ Stokes vector elements were averaged over adjacent pixels by calculating the mean value of each Stokes vector element within a rectangular evaluation window. The DOPU value related to the degree of polarization was then calculated as a function of position. In polarization-preserving or birefringent tissue, DOPU is approximately 1; in the case of a depolarizing layer, DOPU is lower than 1. Red, orange, or yellow colors in DOPU images indicate polarization-preserving tissue (ie, retinal layers other than the RPE). The RPE appears as a depolarizing layer (green to blue colors) providing selective differentiation. By thresholding the DOPU image, areas of DOPU <0.8 were regarded as depolarizing RPE and used to generate an overlay of the segmented RPE and the intensity SD OCT image enabling visualization of retinal layers together with the RPE (Figure 1).

### Intensity-Based Spectral-Domain Optical Coherence Tomography Imaging

Spectralis SD OCT high-resolution macular raster scans, scans of the foveal region, and macular thickness maps were used in this study. Intensity-based SD OCT B-scans were compared to polarization-sensitive OCT images regarding RPE integrity and correlations of hyperreflective material with depolarizing foci in outer retinal layers. Scanning angle was 20 degrees, scan diameter measured 6000 μm, and 20 high-resolution frames were averaged per B-scan.

### Semiautomated Segmentation of Retinal Pigment Epithelium Atrophy by Polarization-Sensitive Optical Coherence Tomography

An automated segmentation algorithm^22^ was developed for GA lesion quantification in dry AMD and used in a semiautomated fashion for measuring lesions in neovascular AMD in this study. The height of the evaluation band used to sum up the number of depolarizing pixels along each A-line was adjusted manually depending on the curvature of the pathologic RPE (ie, in case of pigment epithelial detachment) and on the amount of choroidal depolarizing pixels. Using the evaluation band, the algorithm excluded depolarizing signals below the RPE/Bruch membrane complex and is therefore described as semiautomated. The algorithm analyzes depolarizing pixels along each A-line of a segmented 3D dataset, subsequently generating a thickness map of depolarizing tissue that was then binarized and smoothed to precisely assess GA dimensions. The algorithm automatically detects patches of connected pixels, marks them as atrophic, and calculates the corresponding atrophy area by scaling with the known pixel area.

### Treatment Procedures

Patients received monthly injections of ranibizumab (Lucentis; Novartis Pharma AG, Basel, Switzerland, and Genentech Inc, South San Francisco, California, USA) during the first 12 months and were treated pro re nata (PRN; as needed) from months 12–24 following the Comparison of Age-related Macular Degeneration Treatments Trials (CATT) protocol.^10^ Summarized, patients were scheduled for monthly monitoring visits according to protocol and underwent a complete examination including standardized BCVA testing and SD/polarization-sensitive OCT imaging.

Retreatments were performed if visual deterioration occurred or active CNV was evident. Most retreatment decisions were made according to presence or absence of fluid in OCT. Eyes were retreated except if fluid did not decrease following 3 consecutive monthly injections. Treatment was reinstituted at later visits if increased fluid was detected on OCT. If other signs of active CNV presented (including persistent intraretinal or subretinal hemorrhage, new subretinal hemorrhage, decreased VA relative to last visit without other explanation, leakage on FA, or increased lesion size on FA relative to last angiogram) patients were treated.

A total of 0.5 mg ranibizumab was injected intravitreally using a 30 gauge needle.

### Morphologic Parameters and Data Analyses

Two independent graders analyzed polarization-sensitive and SD OCT images in a masked and random fashion regarding patient, treatment assignment, and visit. Based on polarization-sensitive OCT, RPE-specific changes were graded qualitatively as: RPE thinning, RPE porosity, focal RPE atrophy, GA areas, and regulatory RPE migration including accumulations of depolarizing material at the RPE level or ectopic depolarizing material within outer retinal layers.

**Figure 2:**
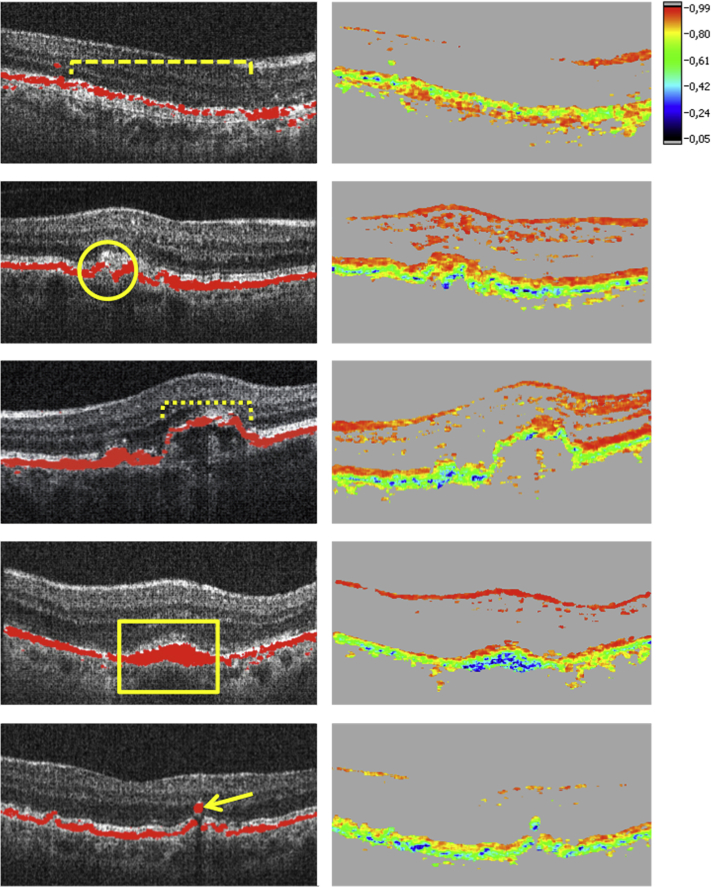
Characteristic changes of the retinal pigment epithelium detected by polarization-sensitive optical coherence tomography in antiangiogenic therapy of neovascular age-related macular degeneration. (Top row) Retinal pigment epithelial (RPE) porosity (dashed brace). (Second row) Focal RPE atrophy (circle). (Third row) RPE thinning (dotted brace). (Fourth row) Thickening of the RPE layer (rectangle). (Bottom row) Ectopic depolarizing material in outer retinal layers (arrow).

RPE porosity was classified as series of RPE layer gaps over a width of at least 20% of a polarization-sensitive OCT B-scan, with several depolarizing RPE residuals between atrophic regions (Figure 2, dashed brace). Focal RPE atrophy was defined as isolated atrophic regions in the RPE, not quantifiable as advanced GA by the segmentation algorithm (Figure 2, circle). RPE thinning was defined as an RPE segmentation band thinner than half the normal RPE segmentation band thickness over a width of at least 10% of a B-scan (Figure 2, dotted brace). GA was defined as atrophic RPE lesions ≥0.1 mm^2^ (minimal GA area detectable by the algorithm).

Accumulation of depolarizing material at the RPE level was defined as an RPE area at least twice as thick as normal RPE identified in a B-scan outside of the AMD lesion (Figure 2, rectangle). Ectopic depolarizing material was specified as depolarizing structures in outer retinal layers, discontinuous from the RPE (Figure 2, arrow).

Quantitative evaluation of presence or absence of described features during follow-up was performed: all polarization-sensitive OCT B-scans were evaluated at each visit and a score of 0 or 1 was assigned for absence (0) or presence (1) of a specific RPE lesion type. The proportion of eyes with evidence of each lesion type was expressed as a percentage for each visit.

Fellow eyes were analyzed and compared to eyes with CNV treated with anti-VEGF based on polarization-sensitive OCT and conventional intensity-based SD OCT. Fellow eyes that developed CNV were immediately treated as needed using intravitreal ranibizumab.

Interobserver variability of qualitative polarization-sensitive OCT results was tested using κ statistics and by calculating percentage of agreement. Spearman correlation coefficients were used to evaluate agreement of identified lesion analyses between observers.

BCVA and central retinal thickness (CRT) (using Spectralis-OCT) were compared during follow-up. The paired sample *t* test was used for BCVA and CRT assessment and for comparison of GA area between different time points. Software used was MedCalc version 14.12.0 (Informer Technologies, Inc.), Excel 2007 (Microsoft), and Photoshop CS5.1 (Adobe). P < .05 was considered statistically significant.

## Results

### Changes in Clinical Parameters

**Figure 3:**
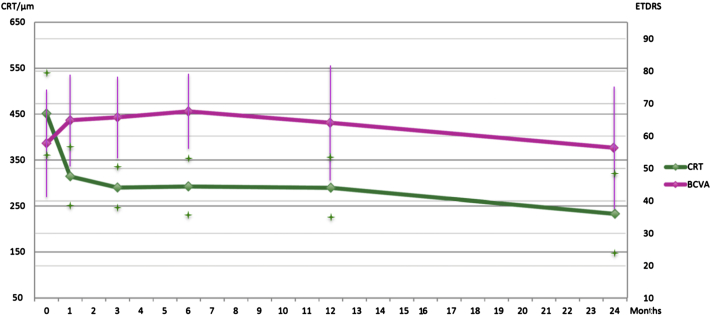
Best-corrected visual acuity and central retinal thickenss values during follow-up in antiangiogenic therapy of neovascular age-related macular degeneration. Compared to month 0 (baseline), statistical significance was *P* = .98 at month 24 for best-corrected visual acuity (BCVA) and *P* < .01 for central retinal thickness (CRT). Bars and stars indicate standard deviation.

At enrollment, BCVA ranged from 20/40 to 20/320. Mean BCVA was 57.7 (range: 14–90) ETDRS letters at baseline and 56.3 (SD: 19.4) letters at month 24 (*P* = .98). Mean CRT was 450.9 μm (SD: 128.6) at baseline and 323.9 μm (SD: 124.8) at month 24 (*P* < .01). Figure 3 summarizes the results. Mean number of anti-VEGF treatments was 9.9 (range: 3–12) between months 0 and 12 and 3.2 (range: 0–6) between months 12 and 24.

### Qualitative Identification of Retinal Pigment Epithelial Morphology by Optical Coherence Tomography Imaging

**Figure 4:**
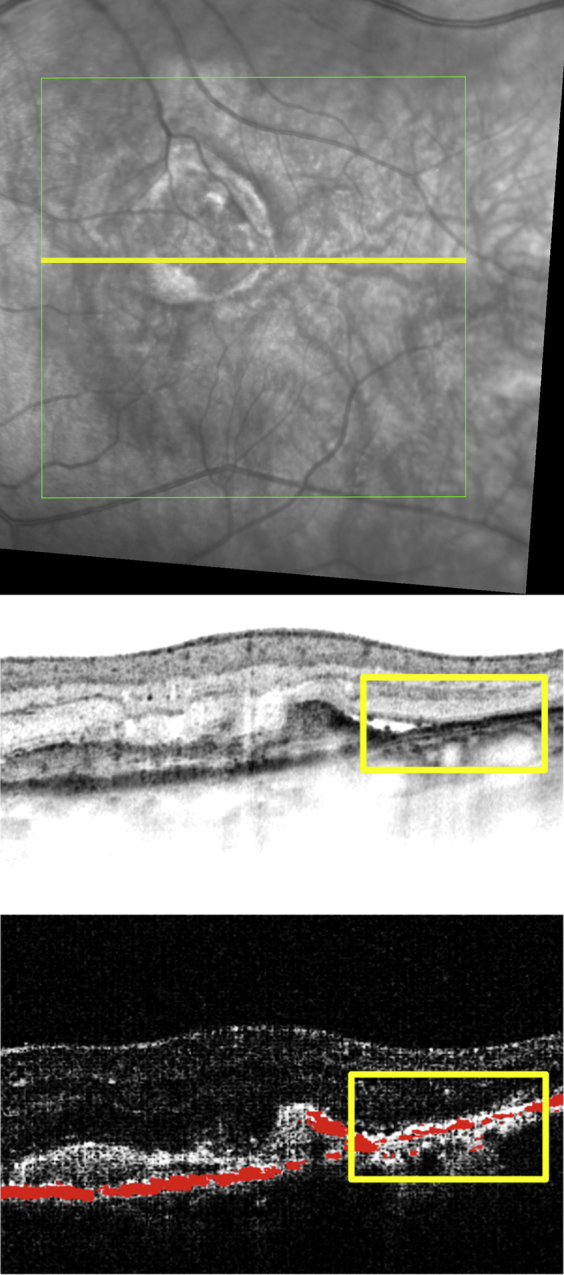
Comparison of polarization-sensitive optical coherence tomography and spectral-domain optical coherence tomography findings in antiangiogenic therapy of neovascular age-related macular degeneration. The position of the respective B-scans is marked (Top); the intensity-based spectral-domain (SD) optical coherence tomography (OCT) image is shown (Middle) and is correlated with the intensity/retinal pigment epithelium (RPE) overlay (Bottom) generated by polarization-sensitive OCT. Severe RPE irregularities were unambiguously detectable by polarization-sensitive OCT in contrast to intensity-based SD OCT, where the condition of the RPE was not identifiable (rectangles). Although an increased light penetration into the choroid is seen in the intensity-based SD OCT image (Middle), associated atrophic RPE and thinned RPE are only unambiguously identifiable using polarization-sensitive OCT (Bottom).

**Figure 5:**
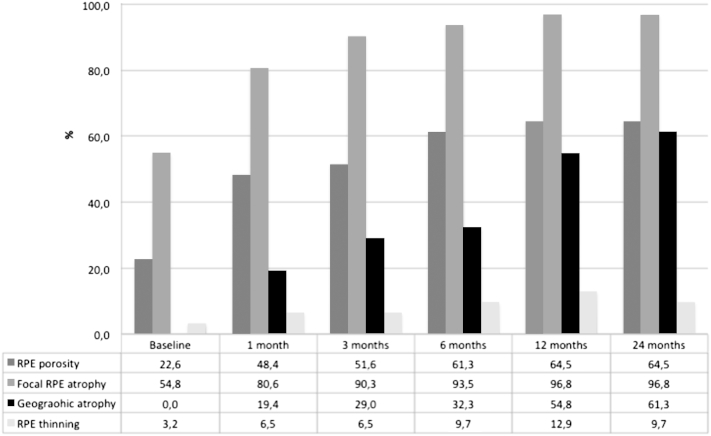
Development of retinal pigment epithelial atrophy under anti–vascular endothelial growth factor treatment in neovascular age-related macular degeneration as detected by polarization-sensitive optical coherence tomography. (Percentages of affected eyes are plotted over time.) Pathognomonic signs of early retinal pigment epithelial (RPE) loss including porosity and focal atrophy increased substantially during the first year of monthly intravitreal treatment and remained stable during the second year with an as-needed retreatment strategy. Areas of geographic atrophy developed over 2 years in 61% of eyes.

**Figure 6:**
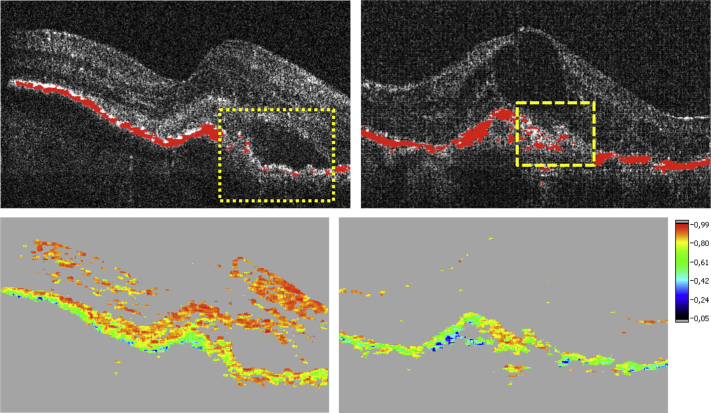
Example of retinal pigment epithelial loss associated with retinal features in antiangiogenic therapy of neovascular age-related macular degeneration. Significant atrophy of the retinal pigment epithelium (RPE) was frequently apparent beneath regions of subretinal fluid (Top; dotted rectangle) or intraretinal cysts (Top; dashed rectangle), specifically detectable by polarization-sensitive optical coherence tomography. (Top) Intensity/RPE overlay; (Bottom) Degree of polarization uniformity. Note that there is sufficient signal penetration present through all retinal layers, allowing for the precise identification of RPE atrophy.

Primary RPE-related alterations caused by the neovascular pathology and/or secondarily associated with anti-VEGF therapy (RPE atrophy and RPE migration) were consistently identifiable by polarization-sensitive OCT (Figure 2). While conventional intensity-based SD OCT demonstrated a continuous RPE layer feature in the majority of cases, polarization-sensitive OCT revealed distinct RPE discontinuities as well as RPE thinning/thickening within the lesion area (Figure 4). At baseline RPE porosity was identified in 23% and focal atrophy in 55% of eyes (Figure 5). RPE atrophy was mostly associated with fluid or intraretinal cysts in SD OCT (Figure 6).

### Progression of Retinal Pigment Epithelial Atrophy in Polarization-Sensitive Optical Coherence Tomography

Continuous progression of RPE loss could be measured during follow-up, based on polarization-sensitive OCT images at each designated visit.

RPE porosity, detected in 7 of 31 eyes (23%) at baseline, increased to 20 affected eyes (65%) at months 12 and 24 during anti-VEGF therapy. Focal RPE atrophy was observed in 17 eyes (55%) at baseline, while following 12 and 24 months 30 eyes (97%) were affected and only 1 eye did not show any area of RPE discontinuity.

**Figure 7:**
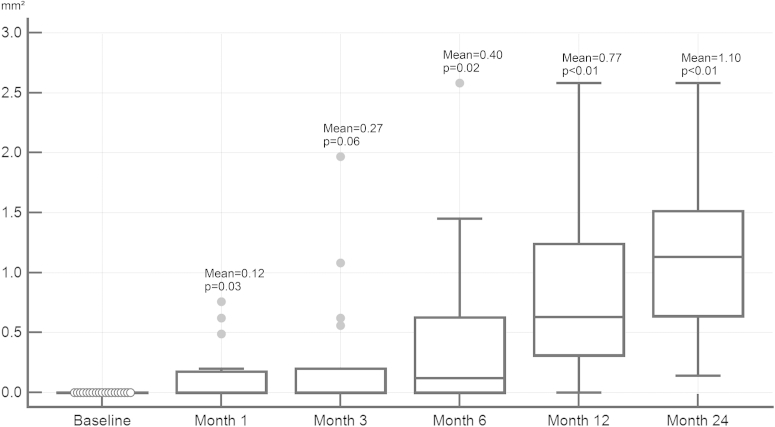
Box-plot analysis of progression in geographic atrophy lesion size during antiangiogenic therapy of neovascular age-related macular degeneration. Although no geographic atrophy (GA) was detectable before treatment, the GA lesion size increased steadily over time. *P* values refer to changes in relation to previous visit. Gray dots refer to outliers. Mean values of GA area are given for each visit.

No GA lesion was detected by the semiautomated segmentation algorithm in any of the 31 eyes at baseline. However, following 12 months of monthly treatment with anti-VEGF, GA was detected in 17 eyes (55%), and in 19 eyes (61%) at month 24. Of all patients in whom GA was detected, mean GA dimensions were 0.77 mm^2^ (SD: 0.67, range: 0.12–2.58 mm^2^) at month 12 and 1.10 mm^2^ (SD: 1.09, range: 0.14–2.58 mm^2^) at month 24 (Figure 7), showing continuous expansion throughout a fixed and a flexible regimen. Eyes with CNV that developed GA showed subretinal drusenoid deposits (SDD) in 5 eyes (26%) at month 24. Twenty-six percent of eyes with CNV developing GA showed SDD prior to the visit when GA was first detected. All eyes with SDD and GA revealed multilobular GA.

**Figure 8:**
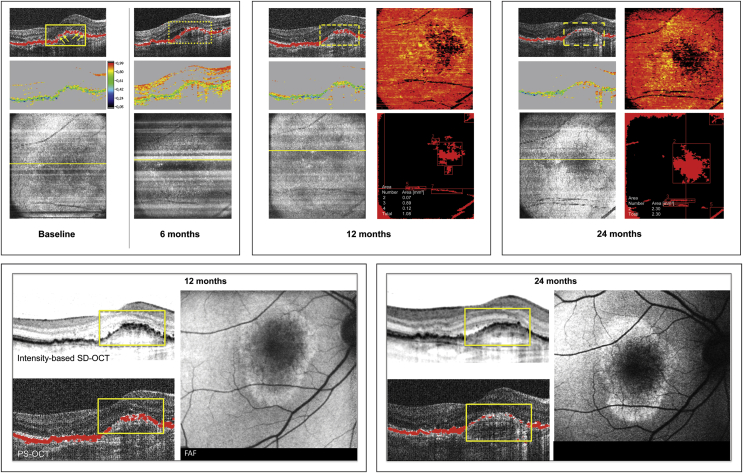
Example of geographic atrophy (GA) progression detected by the semiautomated polarization-sensitive optical coherence tomography (OCT) segmentation algorithm in antiangiogenic therapy of neovascular age-related macular degeneration. Prior to the first injection (baseline) isolated focal retinal pigment epithelial (RPE) atrophy is present (Top left: arrows), progressing in size at month 6 (dotted rectangle in the polarization-sensitive OCT B-scan, Top left). The semiautomated polarization-sensitive OCT segmentation algorithm first detected a distinct GA lesion at month 12 (Top middle: thickness map of depolarizing material, lesion size: 1.08 mm^2^; the dashed rectangle indicates corresponding RPE atrophy in the polarization-sensitive OCT B-scan), progressing to 2.30 mm^2^ at month 24 (Top right: dashed rectangle indicates corresponding RPE atrophy in the polarization-sensitive OCT B-scan). Identical B-scan locations are shown for all time points presented. Intensity/RPE overlay (Top left, Top middle, Top right); degree of polarization uniformity (Top left, Top middle, Top right); pseudo–scanning laser ophthalmoscopy image with marked B-scan position (Top left, Top middle, Top right); RPE thickness map (Top middle, Top right); map indicating increasing GA dimensions over time (Top middle, Top right). The 2 boxes below (Bottom left, Bottom right) represent the identical patient at 12 and 24 months following anti–vascular endothelial growth factor therapy. Note that the status of the RPE is only unambiguously identifiable in the polarization-sensitive OCT B-scans compared to conventional intensity-based spectral-domain OCT imaging. In the fundus autofluorescence images (Bottom left, Bottom right) it is not possible to identify regions of GA and borders of the atrophic zone, whereas the polarization-sensitive OCT maps (Top middle, Top right) specifically identify GA.

Figure 8 shows a representative example of GA detection by polarization-sensitive OCT in a patient treated with anti-VEGF clearly highlighting progressive GA enlargement. Comparing polarization-sensitive OCT with intensity-based SD OCT and FAF imaging, only polarization-sensitive OCT precisely identifies atrophic RPE. Following 24 months of treatment, 9 of 31 eyes (29%) revealed foveal GA, 5 eyes (16%) extrafoveal GA, and 5 eyes (16%) both foveal and extrafoveal GA.

One patient (1 eye, 3%) was affected by RPE thinning at baseline, 4 eyes (13%) at month 12, and 3 eyes (10%) at month 24 following anti-VEGF therapy.

### Evaluation of Retinal Pigment Epithelium Migration Detected by Polarization-Sensitive Optical Coherence Tomography

**Figure 9:**
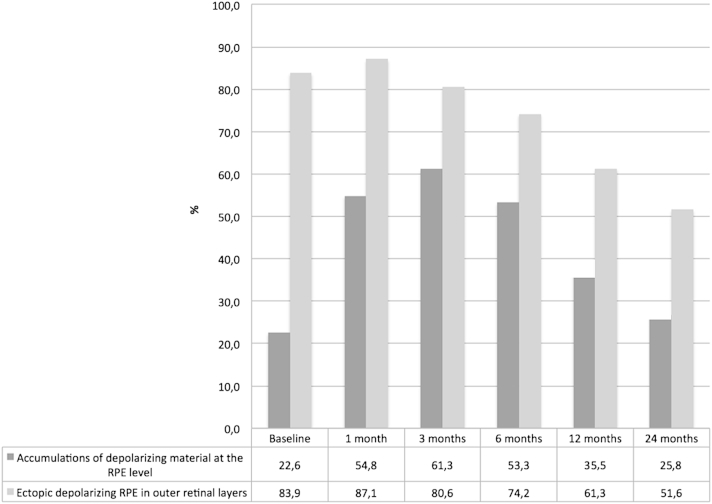
Development of reactive hyperplasia/migration of the retinal pigment epithelium over time detected by polarization-sensitive optical coherence tomography in antiangiogenic therapy of neovascular age-related macular degeneration. The proportion of eyes showing accumulation of retinal pigment epithelium (RPE)-related material at the lesion borders increases until month 3 and subsequently decreases continuously until month 24. RPE-related depolarizing material within outer retinal layers steadily decreases following treatment initiation.

In addition to atrophic changes, an increase in RPE-related material was noted simultaneously and interpreted as reactive RPE migration/hyperplasia, initially increasing until month 3, followed by a decrease of RPE response throughout month 24 (Figure 9).

**Figure 10:**
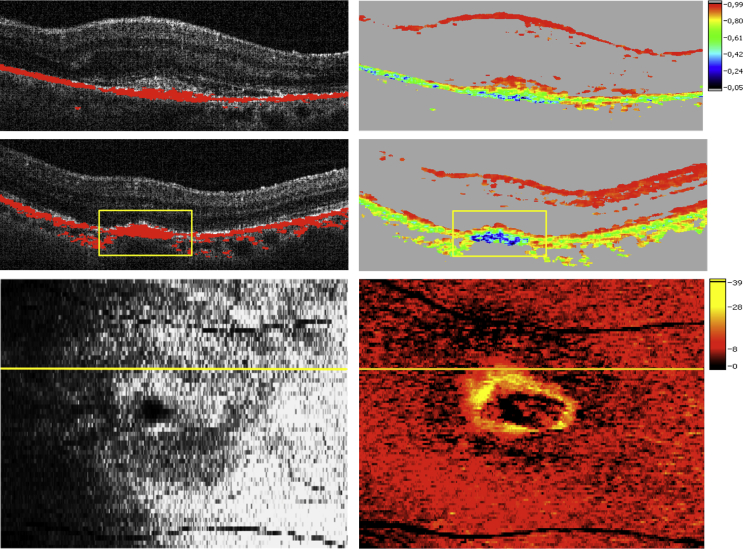
Accumulations of depolarizing retinal pigment epithelium structures detected by polarization-sensitive optical coherence tomography at baseline (Top) and 6 months (Middle, Bottom) following antiangiogenic therapy initiation of neovascular age-related macular degeneration. In its most advanced form, accumulating retinal pigment epithelium (RPE) at the lesion margins demonstrates a ring-shaped appearance surrounding a classic neovascular lesion. Intensity/RPE overlay (Top left, Middle left); degree of polarization uniformity image (Top right, Middle right); pseudo–scanning laser ophthalmoscopy image (Bottom left); thickness map of depolarizing material (Bottom right); note the increased presence of accumulations of depolarizing material at the RPE level at 6 months compared to baseline (rectangles).

Accumulations of RPE-derived depolarization presented in 7 of 31 eyes (23%) prior to treatment, reaching a maximum at month 3 following anti-VEGF therapy (19 eyes, 61%). Eleven eyes (36%) revealed this pattern at month 12 and 8 eyes (26%) at month 24. A typical ring-shaped appearance of accumulating depolarizing material encapsulating the CNV lesion was observed in 3 eyes with classic CNV (Figure 10). All ring-shaped RPE accumulations progressed toward RPE atrophy with treatment continuation.

**Figure 11:**
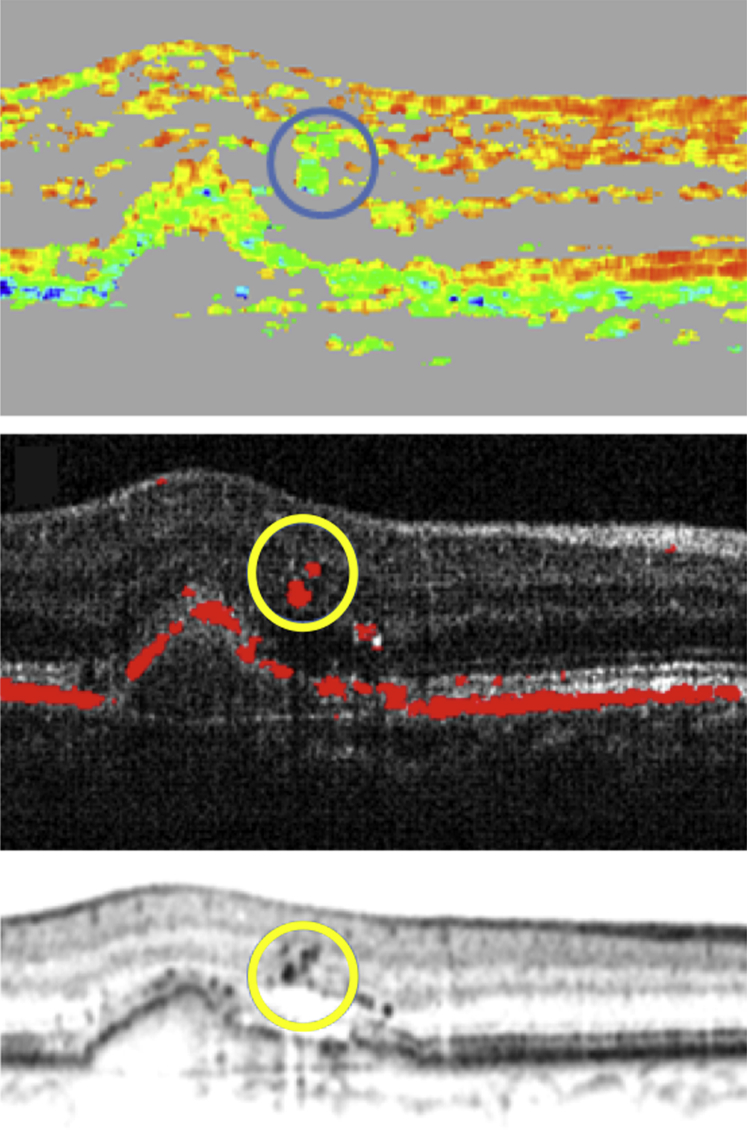
Accumulations of depolarizing material in outer retinal layers in antiangiogenic therapy of neovascular age-related macular degeneration detected by polarization-sensitive optical coherence tomography corresponded to intraretinal hyperreflective accumulations in intensity-based spectral-domain (SD) optical coherence tomography (OCT). The polarization-sensitive OCT degree of polarization uniformity (Top) and intensity/retinal pigment epithelium overlay images (Middle), signifying intraretinal depolarizing material, are illustrated (circle). The intensity-based SD OCT scan (Bottom) reveals corresponding intraretinal hyperreflective material.

Ectopic depolarizing material in outer retinal layers changed from 26 affected eyes (26/31, 84%) at baseline to 19 eyes (19/31, 61%) at month 12 and 16 eyes (16/31, 52%) at month 24. These accumulations were located within the outer nuclear layer (ONL) and consistently corresponded to intraretinal foci in intensity-based SD OCT (Figure 11).

### Morphologic Analysis of Fellow Eyes

Fifteen fellow eyes (48%) developed neovascular AMD until month 24 of follow-up, revealing RPE-related changes as described above, and were therefore excluded from analysis.

Five fellow eyes (31%) showed GA related to dry AMD at baseline, 1 eye (6%) developed GA at month 6. All GA lesions increased progressively until month 24. Drusen presented at a variable extent in all eyes, increasing in number and area in 5 eyes (31%) until month 24. Three fellow eyes (19%) showed drusen regression resulting in focal RPE atrophy or GA until month 24. SDD presented in 13 eyes (81%) at month 24.

In addition to fellow eyes with GA related to dry AMD, 3 eyes (19%) showed focal RPE atrophy, noted at baseline in 2 eyes (13%). These eyes showed isolated focal RPE atrophy on top of a druse, of which 1 eye (6%) remained stable and the other eye showed RPE atrophy progression through month 24. One eye first revealed focal RPE atrophy at month 12 and showed consecutive RPE atrophy progression until month 24. RPE porosity or RPE thinning was not detected in fellow eyes without GA related to dry AMD.

Excluding fellow eyes with CNV, accumulations of depolarizing material in outer retinal layers appeared in 11 eyes (69%) and remained stable in 8 eyes (50%) until month 24. Accumulations of depolarizing material in outer retinal layers increased in 1 of these eyes from months 12–24; 2 eyes showed a decrease from baseline through month 24.

### Reproducibility of Reading Procedures

The Table summarizes interobserver reproducibility of qualitative grading of RPE lesion types in polarization-sensitive OCT B-scans. Highest reproducibility (κ = 1.0) was found for semiautomated GA detection and lowest for RPE thinning (κ = 0.39). Overall agreement was 88%. Semiautomated GA segmentation agreement between observers was consistently high, with r = 0.9 (Spearman correlation coefficient).

## Discussion

This study qualitatively and quantitatively identifies the morphologic response of the RPE during anti-VEGF therapy for neovascular AMD. SD and polarization-sensitive OCT (specifically detecting the RPE) were used to continuously monitor RPE changes before and during anti-VEGF treatment over a follow-up period of 24 months. Characteristic changes of RPE morphology were described, including RPE loss and RPE accumulation.

In this study, an increasing number of eyes showing early RPE atrophy such as RPE porosity occurred with treatment initiation and increased over time, reaching a peak of 65% affected eyes at month 24. Focal RPE atrophy increased with an identical pattern. Compared to GA in dry AMD, presenting with larger RPE atrophy lesions,^25–27^ RPE atrophy in neovascular AMD and associated treatment showed multiple smaller and more diffusely distributed focal atrophic RPE lesions. The multilobular GA structure frequently observed in eyes with CNV of the present study may represent an underlying disease substrate, for example, the choroidal vasculature, recently reported by Xu and associates.^28^ Patients with CNV undergoing therapy frequently developed these discrete RPE discontinuities increasing in size and distribution during follow-up. Jaffe and associates^29^ showed that RPE atrophy other than GA (ie, depigmented RPE without clearly defined boundaries) developed in 15% of eyes under the foveal center in the Comparison of Age-related Macular Degeneration Treatments Trials based on fundus photography and FA. The higher number of patients affected by focal RPE atrophy in our study could be caused by a superior RPE specificity of polarization-sensitive OCT and the fact that these focal RPE lesions were diffusely distributed and not exclusively located subfoveally. Interestingly, localized RPE atrophy was frequently observed beneath intraretinal cysts and fluid accumulation, associated with dysfunctional neurosensory retina.

Recent studies indicated that patients with CNV developed GA^16,29^ following anti-VEGF therapy. Grunwald and associates^16^ reported that 18.3% of patients developed GA within 2 years of anti-VEGF therapy using color fundus photography and FA only. Our study even reveals a linear increase in atrophic RPE features and GA dimensions using polarization-sensitive OCT (61% of GA at month 24). Using the polarization-sensitive OCT-related algorithm, GA was defined as an area ≥0.1 mm^2^ in our study—superior to FA/scanning laser ophthalmoscopy–based detection of GA, commonly defined as an atrophic RPE lesion measuring at least 250 μm in longest linear diameter.^16^ In the current study identification of GA in eyes with CNV was particularly difficult using FAF imaging, whereas polarization-sensitive OCT accurately delineated atrophic RPE (Figure 8). Noteworthy, early atrophic RPE changes (porosity, focal lesions) and expansion of advanced GA was most pronounced during intensive monthly retreatment, consistent with findings in the CATT study and a higher rate of GA in the monthly treatment arm.

Schmitz-Valckenberg and associates^30^ recently showed an association of SDD with GA in dry AMD in 62% of eyes. Our study revealed SDD in only 26% of eyes with GA related to neovascular AMD treated with anti-VEGF at month 24 of follow-up. This supports the hypothesis of an association between GA formation and anti-VEGF therapy that is not linked to progressive dry AMD.

Our findings of progressive RPE atrophy in CNV may therefore indicate a potential complication of VEGF inhibition at the RPE level, in addition to the natural disease course of neovascular AMD primarily causing RPE damage. Although it is not possible to distinguish between the natural disease course and anti-VEGF therapy being the reason for RPE atrophy development, the linear progression over time, the association between intensity of the therapeutic regimen, and the concomitant extent of RPE loss nevertheless support this hypothesis. However, the continuous increase of RPE atrophy detected by polarization-sensitive OCT emphasizes the need for a tight assessment of treatment indication for anti-VEGF therapy in neovascular AMD (avoiding unnecessary therapeutic interventions) to balance benefit and risk. Obviously, although results of our study and other investigations indicate that anti-VEGF therapy may accelerate RPE atrophy in neovascular AMD, treatment is indispensable, otherwise risking progression of disease to legal blindness.

Regarding the reactive response of the RPE to the therapeutic effect (ie, hyperplasia/migration), different patterns were detected by polarization-sensitive OCT in this study, signifying RPE plasticity to some extent. These patterns were generally decreasing until month 24 of follow-up, ending in atrophy. Accumulations of depolarizing structures at the RPE level were most pronounced at month 3, declining toward month 24, which may be consistent with an increase in metabolic activity, dynamic migrating processes, or cellular proliferation, in particular during resolution of exudation in the first 3 months of anti-VEGF therapy. An increase of depolarizing material at the borders of the RPE lesion revealed a topographic correlation to hyperpigmentation in biomicroscopy, supporting the concept of RPE hyperplasia/migration.

Ring-shaped accumulations of depolarizing material at the RPE level surrounding the CNV lesion could indicate an encapsulating, migrating, or extensively proliferating activity of RPE cells at the borders of a regressing CNV lesion, possibly induced by anti-VEGF, described earlier.^31^ A ring-shaped appearance has also been noted in infrared imaging by Semoun and associates,^32^ correlating to the boundaries of the CNV lesion. Our findings of RPE accumulation in polarization-sensitive OCT are further supported by previous in vitro histologic studies, showing RPE recovery accomplished by RPE proliferation and hyperplasia,^33^ signifying RPE recovery in AMD.^34^ Therefore, it may be suggested that the benefit of anti-VEGF therapy could also originate from RPE recovery in cases where RPE migration or proliferation is observable in polarization-sensitive OCT, and not solely from resolution of retinal edema.

The origin of accumulations of depolarizing material in outer retinal layers is unclear, possibly representing exudative components such as previously described lipid exudates^35^ containing melanin from RPE-derived macrophages, migrating into outer retinal layers owing to reactive metabolic activity induced by the neovascular process. These depolarizing foci were typically found at the ONL level and corresponded tightly to accumulations of hyperreflective material in intensity-based SD OCT. This indicates that depolarizing foci in outer retinal layers contain melanin. Histopathological studies would be useful to clarify the precise composition of depolarizing foci. The decreasing amounts of ectopic depolarizing material in outer retinal layers found in our study may also indicate a dynamic response of the RPE following anti-VEGF therapy supported by earlier findings of intraretinal RPE migration in AMD using SD OCT, published by Ho and associates.^36^ The authors described the appearance of intraretinal RPE migration in conventional SD OCT images in 61% of patients with early to intermediate dry AMD. Christenbury and associates^37^ previously showed a positive correlation between hyperreflective foci in SD OCT at baseline and GA development at 2 years of follow-up in intermediate dry AMD. Further studies are needed to determine if depolarizing intraretinal foci are a predictive factor for progression of GA in neovascular AMD.

Fellow eye analysis in our study substantiates an association between short-term progression of RPE changes and therapy, as RPE-related findings described for eyes with CNV were not found in fellow eyes without neovascular AMD.

Regarding BCVA change under therapy, the loss of ≈1 letter at month 24 compared to baseline may be related to the relatively low number of patients included.

A limitation of this study is that the comparison of RPE alterations found in polarization-sensitive OCT B-scans at different visits lacked the implementation of an eye-tracking system that would provide even more accurate results. Fully automated determination of dimensions of all RPE atrophies in a polarization-sensitive OCT dataset will be developed in the future. However, this would require a higher sampling density of polarization-sensitive OCT B-scans and some improvements of the presently used GA segmentation algorithm. Besides large GA lesions, focal RPE atrophy lesions in patients with neovascular AMD are mostly small and diffusely distributed. Furthermore, the semiautomated algorithm for GA detection has limitations in delineating the atrophic zone in cases of severe retinal edema. Nevertheless, ongoing development of software algorithms may soon lead to further studies showing the viability of automatic RPE lesion size detection in neovascular AMD.

Moreover, although previous histologic studies^33,34^ support our hypothesis of increased metabolic activity, migration, or cellular proliferation by adjacent RPE, a direct histopathologic validation cannot be provided in the setting of a clinical study. Experimental studies would be necessary to provide histopathologic correlation analyses.

Although polarization-sensitive OCT does not resolve details within the RPE (ie, at a cellular level), the device images structures like the outer hyperreflective band described earlier^38–40^ based on the concurrent intensity scans, which are part of the SD-based system. This layer may represent the interdigitation of the apical processes of the RPE with cone outer segments, termed “interdigitation zone.”^38^ When assessing polarization-sensitive OCT intensity/RPE overlay images, the segmented RPE frequently masks the outer hyperreflective band because the segmented RPE layer thickness appears wider than one would expect for healthy RPE, consisting of a cell monolayer of a few micrometers in thickness. The reason is a convolution of the real tissue extension with the evaluation window function for RPE detection.^18^ Thickness measurements may be improved by deconvolution, requiring deeper knowledge about statistics of the polarization state distribution caused by the RPE. Furthermore, resolution in DOPU images is lower than in intensity-based images generated by the polarization-sensitive OCT device. Improvements may be achieved by advancing depth resolution by a larger source bandwidth^41^ or by 3-dimensional windowing, requiring a larger sampling density in the y-direction. This would provide an increased number of data points for histogram calculations and/or smaller in-plane window sizes, resulting in better resolution^42^ of the segmented RPE.

In summary, this study revealed essential insights into the morphologic response of the RPE in patients with neovascular AMD treated with anti-VEGF over a follow-up period of 24 months. Based on a novel polarization-sensitive OCT technology, pathognomonic changes could be identified qualitatively and monitored quantitatively. Data strengthen the hypothesis of an increased risk of GA with more intensive VEGF inhibition (ie, monthly retreatment). This knowledge may be useful in routine clinical management, development of future therapeutic strategies, and an improved monitoring of disease and therapy using advanced OCT technologies.

## Figures and Tables

**Table:** Interobserver Agreement Regarding Retinal Pigment Epithelium–Related Changes Identified by Polarization-Sensitive Optical Coherence Tomography During Follow-up in Antiangiogenic Therapy of Neovascular Age-Related Macular Degeneration (Total: 31 Eyes)

		Baseline	Month 1	Month 3	Month 6	Month 12	Month 24	Mean
GA area (automatically detected)	r		0.9	0.9	0.9	1.0	0.9	0.9
		Percentage of Eyes Affected, by Respective Lesion Type						
Focal RPE atrophy	*Grader 1*	54.8	80.7	90.3	93.6	96.8	96.8	
	*Grader 2*	51.6	83.9	93.6	96.8	96.8	96.8	
	*Difference%*	5.9	3.8	3.5	3.3	0.0	0.0	2.8
	κ	0.81	0.80	0.88	0.79	1.00	1.00	0.88
	*95% CI*	0.60–0.91	0.59–0.90	0.75–0.94	0.57–0.90	1.00–1.00	1.00–1.00	
GA (presence per visit and patient)	*Grader 1*	0.0	19.4	29.0	32.3	54.8	61.3	
	*Grader 2*	0.0	19.4	29.0	32.3	54.8	61.3	
	*Difference%*	0.0	0.0	0.0	0.0	0.0	0.0	0.0
	κ		1.00	1.00	1.00	1.00	1.00	1.00
	*95% CI*		1.00–1.00	1.00–1.00	1.00–1.00	1.00–1.00	1.00–1.00	
RPE porosity	*Grader 1*	22.6	48.4	51.6	61.3	64.5	64.5	
	*Grader 2*	29.0	48.4	54.8	67.7	71.0	54.8	
	*Difference%*	22.2	0.0	5.9	9.5	9.1	15.0	10.3
	κ	0.91	0.85	0.89	0.93	0.93	0.81	0.89
	*95% CI*	0.82–0.95	0.69–0.92	0.78–0.95	0.85–0.97	0.85–0.96	0.61–0.91	
Accumulations of depolarizing material at the RPE level	*Grader 1*	22.6	54.8	61.3	53.3	35.5	25.8	
	*Grader 2*	29.0	58.1	61.3	54.8	41.9	32.3	
	*Difference%*	22.2	5.5	0.0	2.8	15.3	20.2	11.00
	κ	0.91	0.89	0.93	0.97	0.85	0.91	0.91
	*95% CI*	0.82–0.96	0.78–0.95	0.85–0.97	0.93–0.98	0.69–0.93	0.83–0.96	
RPE thinning	*Grader 1*	3.2	6.5	6.5	9.7	12.9	9.7	
	*Grader 2*	6.5	0.0	9.7	6.5	16.1	9.7	
	*Difference%*	49.9	100.0	33.4	33.4	20.0	0.0	39.5
	κ	0.79	0	0.26	0.88	0.52	0.42	0.39
	*95% CI*	0.57–0.90	−1.07 to 0.52	−1.62 to 0.39	0.76–0.94	0.01–0.77	−0.21 to 0.72	
Ectopic depolarizing material in outer retinal layers	*Grader 1*	83.9	87.1	80.7	74.2	61.3	51.6	
	*Grader 2*	83.9	90.3	80.7	71.0	38.7	35.5	
	*Difference%*	0.0	3.6	0.0	4.3	36.8	31.3	12.7
	κ	1.00	0.92	1.00	0.96	0.77	0.80	0.91
	*95% CI*	1.00–1.00	0.82–0.95	1.00–1.00	0.92–0.98	0.53–0.89	0.59–0.91	

CI = confidence interval; GA = geographic atrophy; κ = intraclass correlation coefficient; RPE = retinal pigment epithelium. The Spearman correlation coefficient of semiautomatically quantified GA lesions between both observers was r = 0.9. Overall agreement was 88% between both graders regarding the assessment of RPE-specific lesions detected in polarization-sensitive optical coherence tomography B-scans.
